# Seismic performance evaluation of precast post-tensioned high-performance concrete frame beam-column joint under cyclic loading

**DOI:** 10.1038/s41598-024-63083-y

**Published:** 2024-05-29

**Authors:** Xuefei Pang, Yangchun Li

**Affiliations:** College of Architecture and Engineering, Zhanjiang University of Science and Technology, Zhanjiang, 524000 China

**Keywords:** Post-tensioned precast concrete, Seismic performance, Residual deformation, RPC, Numerical analysis, Natural hazards, Engineering

## Abstract

Precast concrete structures have developed rapidly because they meet the requirements of green and low-carbon social development. In this paper, a precast post-tensioned high-performance concrete frame beam-column joint was proposed, and the low-cycle reversed load test was performed on the four proposed joints. The main differences between the four joints are the different prestress values applied by the joints and whether the beam-column joint is provided with L-shaped steel. The seismic performance indexes such as hysteresis curve, stiffness degradation, deformation capacity, energy dissipation capacity and residual deformation of each node were obtained through experiments. By comparing various seismic performance indicators, it could be found that the use of high-performance concrete could effectively avoid the phenomenon of local crushing of concrete due to excessive prestressing. At the same time, it was found that the setting of L-shaped steel plate at the beam-column junction could effectively avoid the early damage at the beam-column junction. On the basis of the test, the three-line restoring force model of the joint was established by the method of experimental regression analysis. The model could better reflect the stress situation of each stage of the joint. Based on the experimental and theoretical analysis, the finite element analysis model of the joint was established, and the model calculation results were in good agreement with the experimental results.

## Introduction

Precast concrete structure has a history of more than 140 years since it emerged in Europe at the end of the nineteenth century^1^. In this process, precast concrete structures are increasingly used worldwide because of their advantages in construction quality, construction efficiency, and material savings, reducing energy consumption and labor, reducing emissions and pollution^2,3^. Vigorously developing prefabricated buildings, especially prefabricated buildings with prefabricated concrete structures, plays an important role in promoting the transformation and upgrading of the traditional construction industry. Precast concrete frame structure and precast concrete shear wall structure are the most important structural systems in precast concrete structures^4–6^. Precast concrete frame structure is widely used in public buildings, residential buildings and industrial buildings because of its flexible interior space layout and rich building facades.

At present, many scholars have done a lot of research on the assembled concrete frame structure. In 1986, Park et al.^7^ conducted low-cycle reversed loading tests on three full-scale models of beam-column exterior joints. The results show that the beam-column joints of such precast concrete frame structures have good seismic performance. Xue et al.^8–10^ through the full-scale model test of beam-column joints. The failure mode, failure mechanism, bearing capacity, ductility, energy dissipation capacity and stiffness degradation law of this kind of precast concrete frame structure are basically consistent with those of cast-in-place frame structure. Chen et al.^11^ and Liu et al.^12^ completed a series of low cyclic loading tests on full-scale models of frame beam-column joints. Alcocer et al.^13^, Ertas et al.^14^, Im et al.^15^, Eom et al.^16^ and Yuksel et al.^17^ performed full-scale beam-column joint model low cyclic loading tests. The results show that the integrity and seismic performance of beam-column joints with multi-layer prefabricated columns are better, and the bearing capacity and ductility are similar to those of the corresponding cast-in-place joints. With the continuous development of high-strength concrete and high-strength steel, the application of two high-strength materials in precast concrete frame structures has also received more and more attention. Zhao et al.^18^ carried out low cyclic loading tests on four full-scale models of precast column-superimposed beam frame joints using high-strength concrete and high-strength steel fiber reinforced concrete. The hysteretic curves of this kind of precast concrete frame joints are similar to those of cast-in-place frame joints. Liu et al.^19^ carried out a low cyclic loading test on a full-scale model of precast concrete frame beam-column joints with large diameter and large spacing HRB500 high-strength steel bars. The results show that the precast concrete frame beam-column joints have good seismic performance, and the displacement ductility coefficient is 4.5–5.6. Yu et al.^20^ employed the prestressed steel strands as the force bar at the bottom of the precast beam, so that the flexibility of the steel strand can be used to avoid the collision of the force bars in the joint area during the beam-column assembly. Based on the previous research, Yu et al.^21^ proposed to use RPC to pour in the joint area and the keyway area to improve the strength of the joint area and the keyway area and prevent the local area from being crushed in advance.

**Figure 1:**
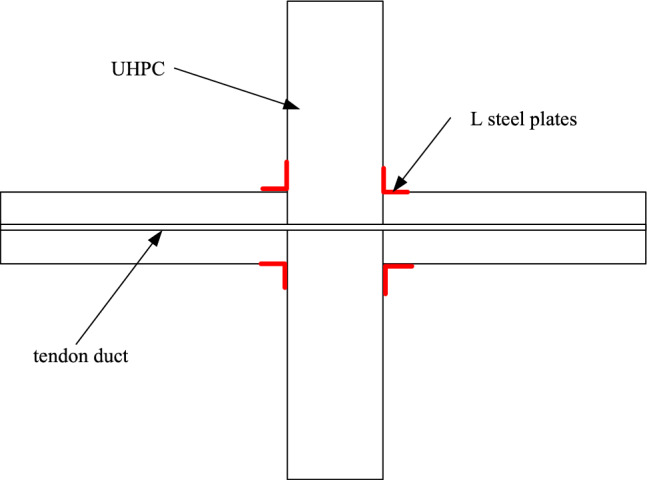
Structure diagram of joint.

The joint proposed in this paper has the following characteristics: (1) The concrete used in the precast beam and precast column is RPC, and its strength can reach more than 100 MPa, which can effectively improve the ultimate bearing capacity of the joint. (2) The post-tensioned prestressing is used to splice the prefabricated beams and columns, which reduces the wet operation and improves the assembly efficiency. (3) The L-shaped steel plate is set at the beam-column junction, which can enhance the energy dissipation capacity of the joint, and can also enhance the strength of the beam-column joint surface, and effectively protect the beam-column joint surface from early damage. The joint construction diagram is shown in Fig. 1.

## Experimental research

### Design and details of the specimens

**Figure 2:**
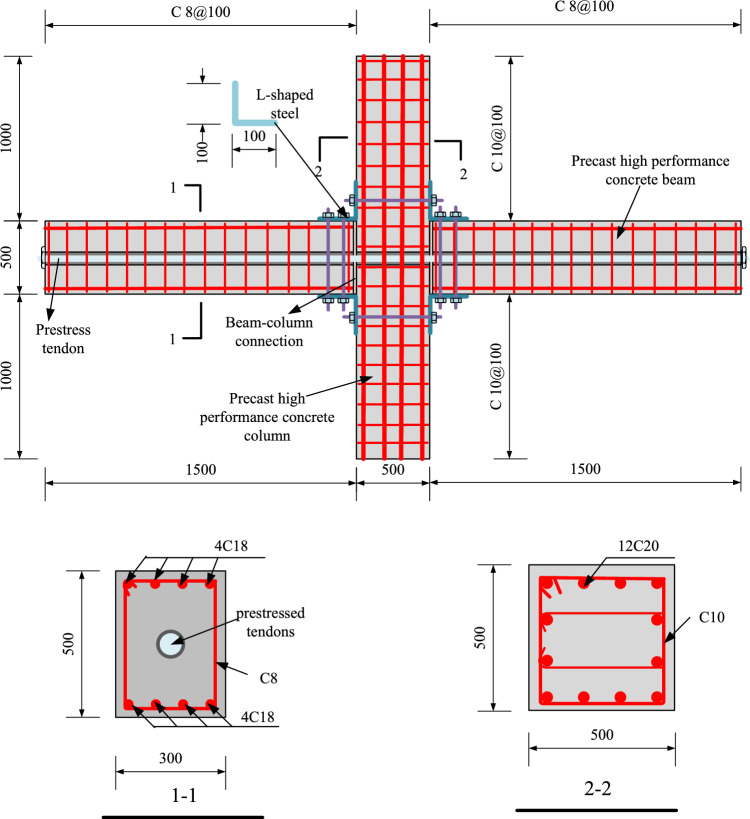
Design details of joints (mm). Note: C = HRB400, HRB400 represents steel bars with a strength grade of 400 MPa.

In this test, four post-tensioned prestressed assembled beam-column joints were designed and numbered PC1–PC4. The main difference between the two joints is the different prestress applied and whether L-shaped steel is set at the beam-column junction, The details of the joints are shown in Table 1. The effects of prestress and L-shaped steel on the seismic performance of joints were studied by experiments. The concrete used in the two joints is reactive powder concrete (RPC), and its strength can reach more than 100 MPa. The cross-sectional dimensions of the four specimens are the same. The cross-sectional dimension of the precast column is 500 × 500 mm, and 12 longitudinal reinforcements with a diameter of 20 mm are configured. The stirrup spacing of the column is 10 @ 100. The section size of the precast beam is 300 × 500 mm. The upper and lower stress bars are all four steel bars with a diameter of 18, and the stirrup is 8 @ 100. The steel bars used in this specimens are HRB400 (strength grade 400 MPa). The RPC strength grade used in this precast beam and precast column is 100 MPa. Details of the joint design are shown in Fig. 2.

**Table 1:** Specimen details.

Specimen number	Setting up L-shaped steel	Prestress of tendons (MPa)
PC1	Setting	1080
PC2	Setting	870
PC3	Not set	1080
PC4	Not set	870

### Materials properties

The RPC is mainly composed of ordinary Portland cement with a strength grade of 52.5 MPa, quartz sand, steel fiber, silicon powder, superplasticizer and water. The mix proportion of RPC is shown in Table 2.

**Table 2:** Mix proportion of RPC.

Material	Cement	Slica fume	Quartz sand	Steel fiber	Superplasticizer	Water
Quantity (kg/m^3^)	756.7	227	1278	105.6	20.5	168.7

**Figure 3:**
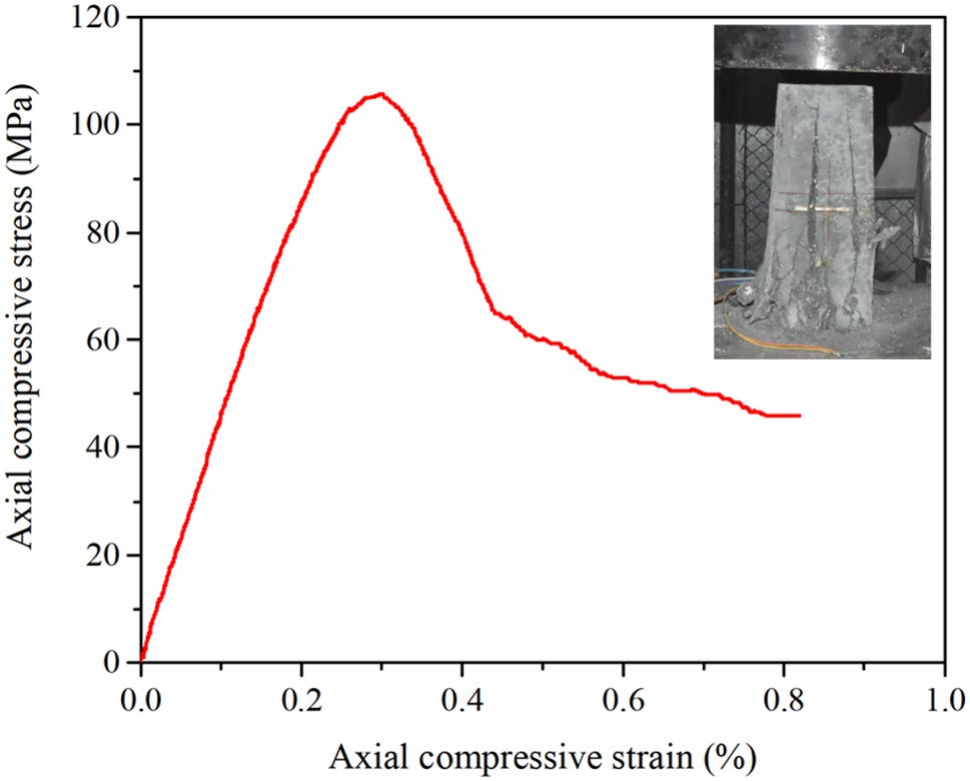
Compressive stress–strain curve.

**Figure 4:**
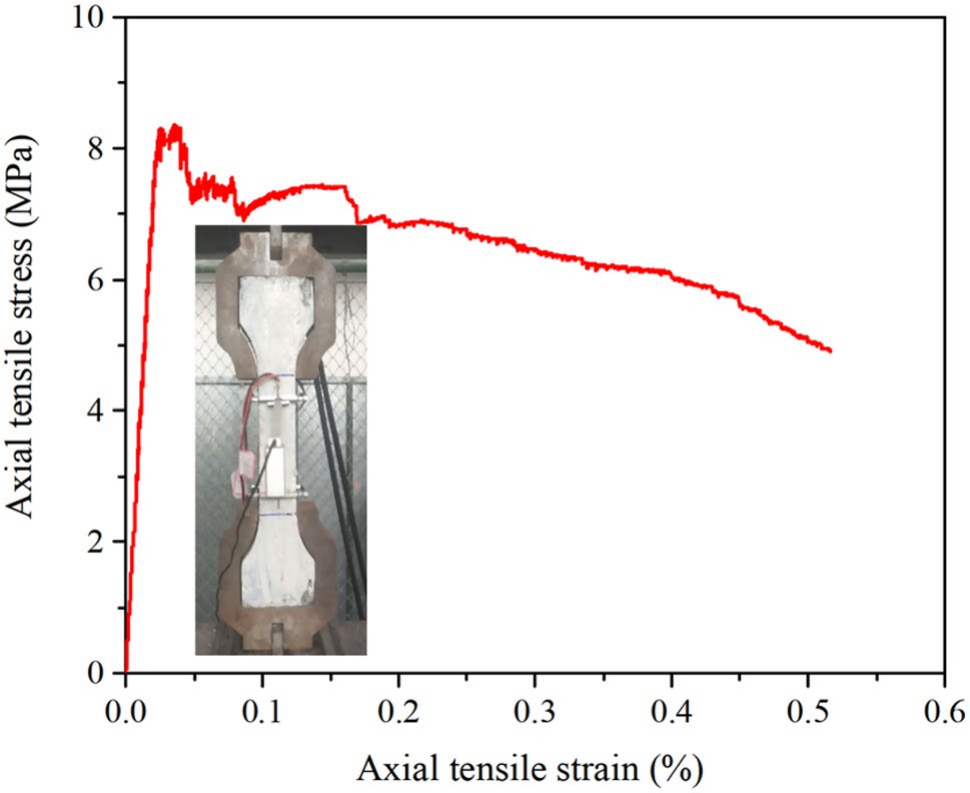
Tensile stress–strain curve.

According to the Chinese standard 'CECS13-2009 Fiber Reinforced Concrete Test Method Standard'^22^, the compressive stress–strain curve of RPC is obtained by compression tests on three prisms with a cross-sectional dimension of 100 mm × 100 mm × 300 mm, as shown in Fig. 3. The axial compressive strength and elastic modulus of RPC are 105.5 MPa and 46.1 GPa, respectively. The tensile stress–strain curve of RPC is shown in Fig. 4. At the same time, the material properties of steel bars and steel strands were tested. Three specimens were tested for each steel bar. The test results are shown in Table 3.

**Table 3:** Mechanical behavior of reinforcing bars.

Steel specifications	Specimens	Diameter (mm)	Yield strength (MPa)	Ultimate strength (MPa)	Modulus of elastic (GPa)
HRB400	Column reinforcement	20	443.4	635.3	200
HRB400	Beam reinforcement	18	430.6	625.8	200
HRB400	Column stirrup	10	441.7	631.7	200
HRB400	Beam stirrup	8	425.6	623.4	200
Strands	Prestress bars	12.7	1620	1927	195

### Test setup and loading procedure

**Figure 5:**
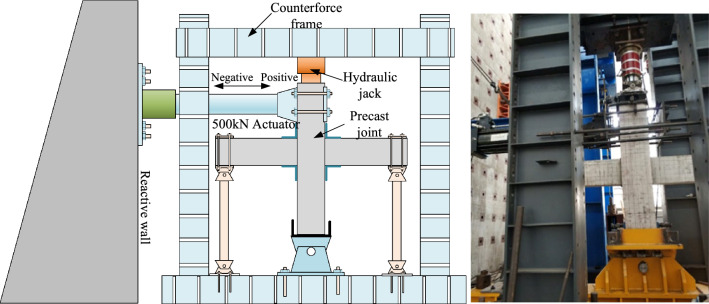
Loading equipment.

**Figure 6:**
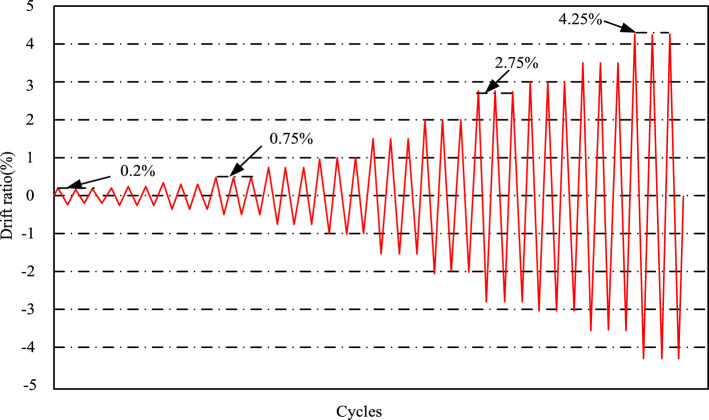
Loading system.

The test loading device is shown in Fig. 5. The beam end is supported by a vertical roller, and the bottom of the column is supported by a pin shaft. The height from the loading point on the upper part of the column to the pin shaft at the bottom of the column is 2650 mm. A fixed axial load is applied to the top of the column to ensure that the axial compression ratio of the column is 0.2, the axial compression ratio of 0.2 is determined according to the loading device. According to ACI 374.-05^23^, the actuator with a range of 1500 kN is used to apply low-cycle repeated load to the joint, and the load cycle is 3 times per stage, the specific loading system is shown in Fig. 6.

## Experimental results and analysis

### Crack pattern and failure mode

**Figure 7:**
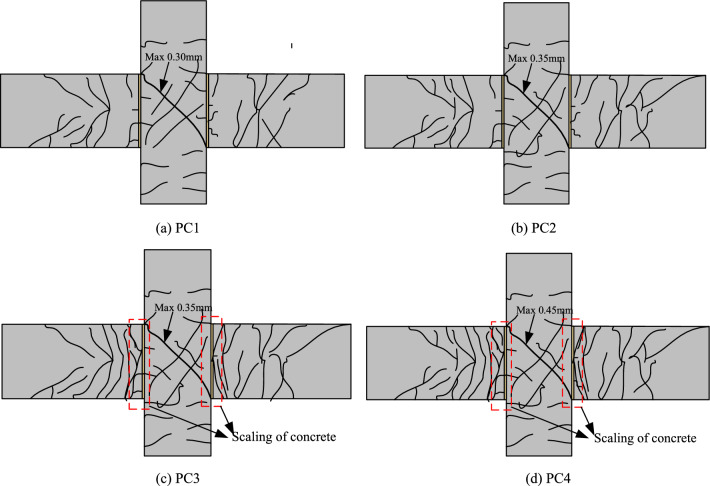
The failure pattern of joints.

Under the action of low cyclic loading, the cracking process and characteristics of the specimen are basically similar, which mainly experience the following three stages: In stage 1, vertical cracks or oblique cracks first appear in the plastic hinge area of the beam end. With the increase of displacement, the original cracks continue to extend and new cracks continue to appear and develop away from the core area of the joint. In stage 2, diagonal cracks appear in the core area of the joint. When reverse loading is applied, cross diagonal cracks are formed. As the load increases, diagonal cracks parallel to the first crack appear, and the length and number of cracks increase continuously. In stage 3, the main diagonal crack of the joint is widened, and the crack width continues to increase as the displacement increases. Figure 7 shows the final failure modes of two specimens.

Through the final failure modes of the four joints, it can be found that the damage degree of the joints PC1 and PC2 with L-shaped steel is significantly lighter than that of the joints PC3 and PC4. This is mainly because the setting of L-shaped steel effectively enhances the strength of the beam-column joint and avoids serious damage under low cyclic loading. At the same time, compared with PC1 and PC2 nodes, it can be found that because the prestress value applied by PC1 node is larger than that of PC2, the final crack of PC2 node is more than that of PC1 node, and the maximum crack in the node area is wider than that of PC1. The PC3 and PC4 joints were seriously damaged at the beam-column junction, and the concrete spalling occurred. At the same time, it can be found that due to the large prestress value applied to the PC3 joint, the crack development is less than that of the PC4 joint.

### Hysteresis characteristics

**Figure 8:**
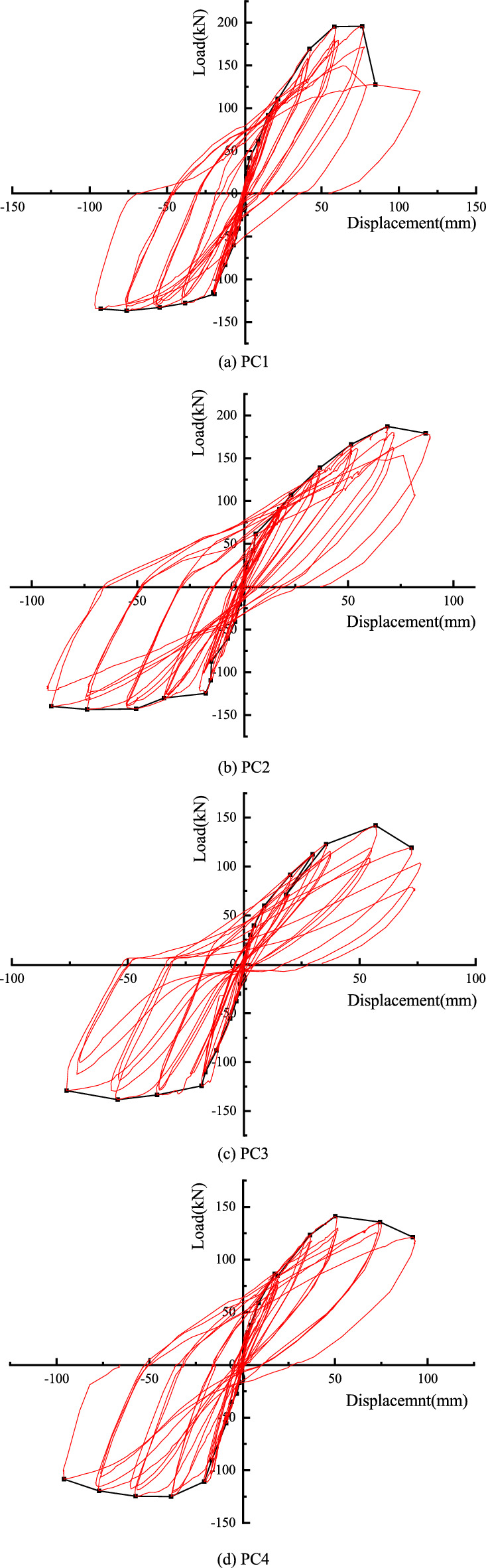
Hysteresis characteristics.

The hysteresis curves of the four joints are shown in Fig. 8. It can be found that the four joints have experienced three stages of elasticity, yield and failure. In the early stage of loading, the four nodes are in the elastic stage, and the hysteresis curves are basically linear. The area of the hysteresis loop is very small, and there is no residual deformation. As the displacement load continues to be applied, the joints show geometric nonlinearity, and the joints are approximately in a nonlinear elastic state. PC1 and PC2 joints are further applied with the loading displacement, the L-shaped steel begins to yield, the beam body also begins to crack, the energy dissipation capacity of the node is enhanced. With the continuous development of cracks, the yield of L-shaped steel and the continuous development of beam cracks, the material nonlinearity of joints gradually appears, and the stiffness of joints further decreases and tends to be stable. With the increase of the deformation of the energy dissipation angle steel, the hysteresis curve becomes more and more full. For the joints PC3 and PC4, there is no L-shaped steel at the beam-column junction, and there is also the effect of prestress. It can be found that the hysteresis curves of the two joints are pinched compared with PC1 and PC2 joints. It can be seen that setting L-shaped steel at the beam-column junction is beneficial to improve the fullness of the hysteresis loop of the joint, thereby improving the hysteretic energy dissipation capacity of the joint. Setting L-shaped steel at the beam-column junction is also beneficial to improve the bearing capacity of the joints. It can be seen that the bearing capacity of the joints PC1 and PC2 with L-shaped steel is much higher than that of the joints PC3 and PC4. Through the hysteresis curve, it can also be seen that the residual deformation of the four nodes before the failure is small due to the prestressing of the four nodes.

### Stiffness degradation

The secant stiffness under each cycle can be expressed as follows^24^:

1 $$K_{i} = \frac{{\left| { + F_{i} } \right| + \left| { - F_{i} } \right|}}{{\left| { + X_{i} } \right| + \left| { - X_{i} } \right|}}$$

In the formula, $+ F_{i}$, $- F_{i}$—The load value of the ith positive and negative peak point; $+ X_{i}$, $- X_{i}$—The displacement of the ith positive and negative peak point.

**Figure 9:**
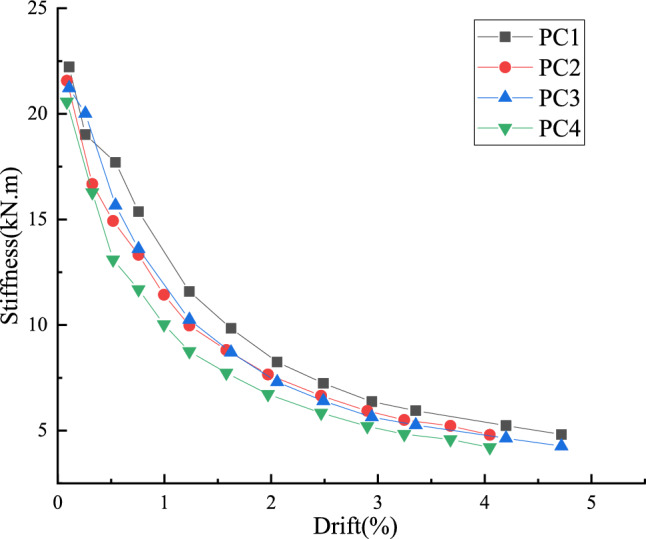
Stiffness degradation.

The stiffness degradation of the four post-tensioned prestressed fabricated joints is shown in Fig. 9. With the increase of loading displacement, the cracks of beam-column joints continue to develop, the plastic deformation of L-shaped steel accumulates, the cracks of beam develop, and the stiffness of joints decreases with the increase of displacement. With the generation and development of the gap at the beam-column joints, the stiffness of each specimen decays rapidly at the initial stage of loading. The prestress value of PC2 is larger than that of PC1. From Fig. 9, it can be seen that the initial stiffness of the four nodes is roughly the same. With the increase of loading displacement, the stiffness value of PC1 joint is always greater than that of the other three joints due to the large prestress and the L-shaped steel is set at the beam-column joint. Because the L-shaped steel plate has not participated in the work in the early stage of loading, the stiffness value of the PC2 joint is slightly smaller than that of the PC3 joint with larger prestress. However, as the load is applied, the L-shaped steel begins to participate in the work, and its stiffness value is basically the same as that of the PC3 joint. The prestress applied to the PC4 node is small, and the L-shaped steel is not set, and the stiffness value is the smallest. From the above analysis, it can be seen that the initial stiffness of the joint mainly depends on the stiffness of the beam and column itself, and has nothing to do with the initial stress of the prestressed tendons. The stiffness of the joint after the limit state mainly depends on the stiffness of the beam and column itself, the stiffness contributed by the prestress and the stiffness contributed by the L-shaped steel.

### Load capacity

**Figure 10:**
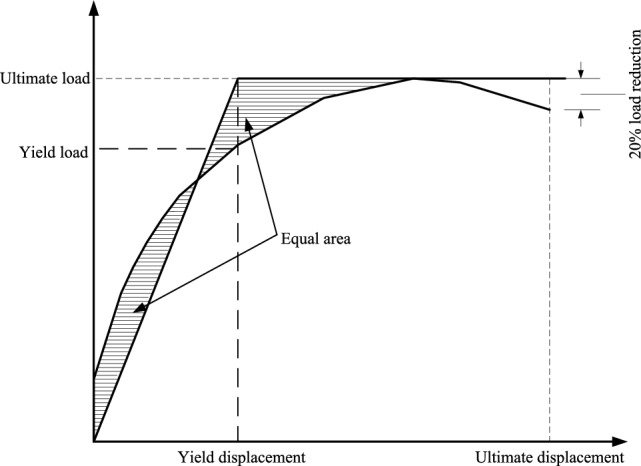
Equal area method diagram.

The equivalent area method has a relatively clear physical meaning and is widely accepted to determine the yield point^24^. In this study, the area of the equivalent two-fold line is equal to that of the original curve envelope. The point on the original curve corresponding to the equivalent bilinear turning point is considered to be the yield point. The schematic is shown in Fig. 10.

Yield load and ultimate load are listed in Table 4. Through Table 4, it can be found that the ultimate bearing capacity of PC1 joints with high prestress and L-shaped steel is the highest, which is 45.7% higher than that of PC4 joints. The deformation capacity of PC3 is the worst, and its ultimate displacement is weaker than that of the other three nodes. The main reason is that the prestress value applied by PC3 is larger, and the concrete at the beam-column joint is crushed in advance. For PC1 and PC2 joints, the L-shaped steel is set at the beam-column joint, and the deformation capacity is strong during the loading process, due to the fear of excessive displacement loading, the failure of the anchorage end of the prestressed tendon occurs, the loading of the node is stopped, and the actual node can continue to bear the load. This also shows that the prestressed assembly joint has a good safety reserve.

**Table 4:** The carrying capacity of the joints.

Joints	Direction	Yield load (kN)	Yield displacement Δ_u_ (mm)	Peak load (kN)	Ultimate displacement Δ_y_ (mm)	Yield ratio	Ductility factor Δ_u_/Δ_y_
PC1	Forward	105.32	30.65	201.7	96.25	1.92	3.32
	Reverse	− 83.20	26.70	− 147.23	− 93.45	1.78	
PC2	Forward	96.33	26.45	189.8	90.45	1.97	3.98
	Reverse	− 78.51	19.92	− 145.21	− 87.67	1.85	
PC3	Forward	87.23	34.50	147.6	81.76	1.69	2.76
	Reverse	− 74.21	25.60	− 136.21	− 80.63	1.83	
PC4	Forward	80.38	33.17	138.46	95.21	1.72	3.16
	Reverse	− 70.35	27.03	− 128.7	− 93.25	1.83	

Δ_u_—Yield deformation of specimen; Δ_y_—Ultimate deformation of specimen.

### Energy absorption

**Figure 11:**
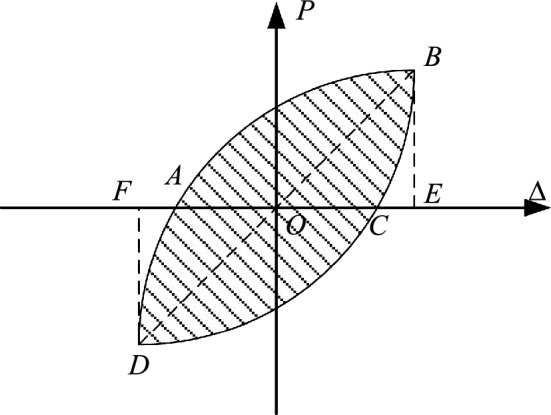
Energy dissipation coefficient calculation diagram.

The energy dissipation capacity of the joint is obtained according to the area enclosed by its hysteresis curve. The change of cumulative energy dissipation with displacement is shown in Fig. 11. The energy dissipation coefficient E can be calculated as follows^24^:

2 $$E = \frac{{S_{{\left( {ABC + CDA} \right)}} }}{{S_{{\left( {OBE + ODG} \right)}} }}$$

$S_{{\left( {ABC + CDA} \right)}}$—The area surrounded by hysteresis loops in Fig. 11; $S_{{\left( {OBE + ODG} \right)}}$—The area of triangle OBE and ODG in Fig. 11.

**Figure 12:**
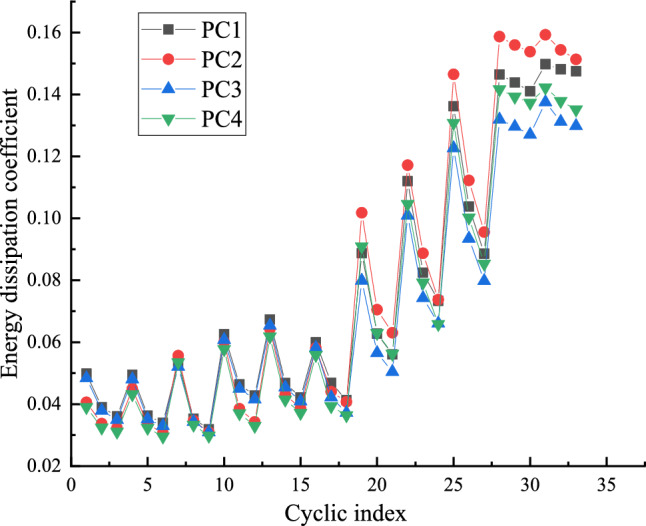
Energy dissipation coefficient of joints.

The energy dissipation coefficient of each specimen is shown in Fig. 12. Obviously, at the initial stage of loading, the four specimens have little energy consumption due to the nodes are in the elastic stage. However, with the increase of loading displacement, in the yield stage, the viscous damping coefficients of the four joints show a rapid upward trend. Prestress degree λ of precast joint is defined as the ratio of resistance moment provided by prestressed tendon to resistance moment provided by prestressed tendon and angle steel. Through Fig. 12, the energy dissipation capacity of PC1 and PC2 joints with L-shaped steel is better than that of PC3 and PC4 joints. However, because the prestressing degree of PC1 joints is greater than that of PC2 joints, the energy dissipation capacity of PC2 joints with smaller prestressing degree is better than that of PC1 joints after the specimen yields. The prestress degree λ is small, the pinching effect of the prestressed tendons is reduced, and the energy dissipation capacity of the node is enhanced.

### Residual displacement

**Figure 13:**
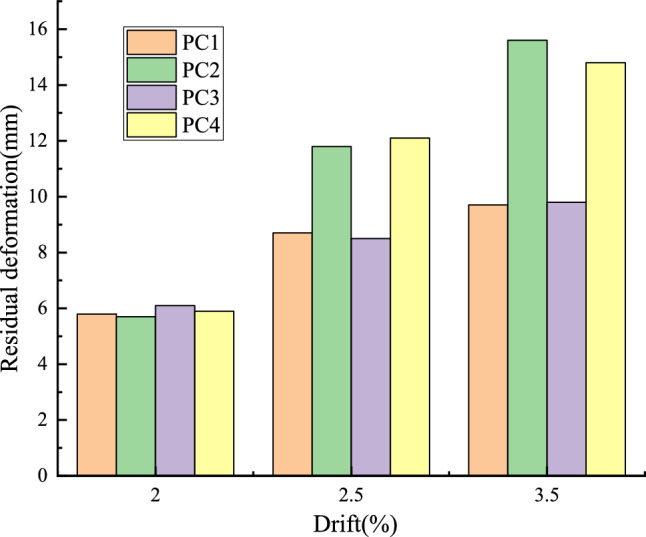
Residual deformation of joints.

Figure 13 shows that the residual deformation of four joints is basically the same when the drift ratio is 2%, but as the load continues to be applied, when the drift ratio is applied to 2.5%, It can be found that the residual deformation of nodes PC1 and PC3 with larger prestress values is smaller than that of nodes PC2 and PC4. When the load is applied to the 3.5% drift ratio, the difference of residual deformation between the four nodes is more obvious. The residual deformation of PC1 and PC3 nodes is much smaller than that of PC2 and PC4 nodes. However, it does not mean that the greater the prestress is applied, the smaller the residual deformation of the structure. When the prestress is applied, it is necessary to fully consider whether the prestress applied will cause the prefabricated components to be crushed locally in advance and directly enter the failure stage of the structure. Therefore, in order to better restore the deformation of the unloading stage, it is necessary to reasonably design the size of prestressed and confined steel bars considering that the concrete is not crushed.

**Figure 14:**
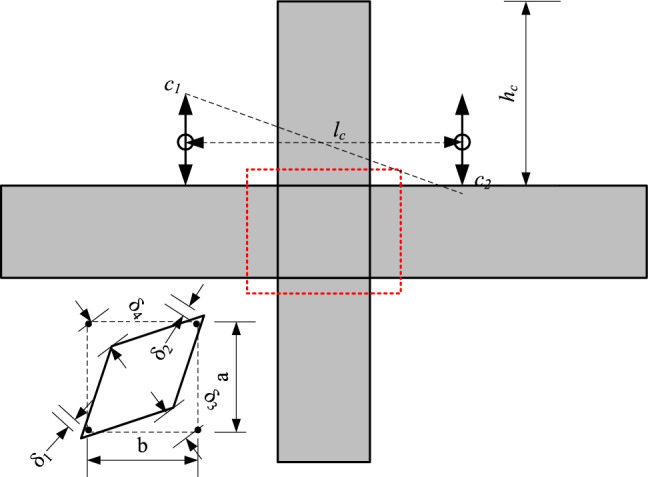
Column top displacement calculation diagram.

For frame joints, the displacement of column top is mainly composed of column bending deformation, shear deformation of joint core area, bending deformation and slip of beam end. As shown in Fig. 14, the displacement of column top can be expressed as:

3 $$\delta = \delta_{b} + \delta_{c} + \delta_{core}$$

In the formula, δ—Column top displacement; δ_*b*_—The displacement of the loading end caused by the bending, slip and shear deformation of the beam end; δ_*c*_:—The displacement of the loading end caused by the bending deformation of the column end; δ_*core*_—Loading end displacement caused by shear deformation of core region.

4 $$\delta_{b} = \frac{{c_{1} + c_{2} }}{{l_{c} }} \cdot h_{c}$$

5 $$\delta_{core} = \frac{{\sqrt {a^{2} + b^{2} } }}{ab} \cdot \frac{{\left| {\left( {\delta_{1} + \delta_{2} } \right)} \right| + \left| {\left( {\delta_{3} + \delta_{4} } \right)} \right|}}{2} \cdot h_{c}$$

6 $$\delta_{c} = \delta - \delta_{b} - \delta_{core}$$

**Figure 15:**
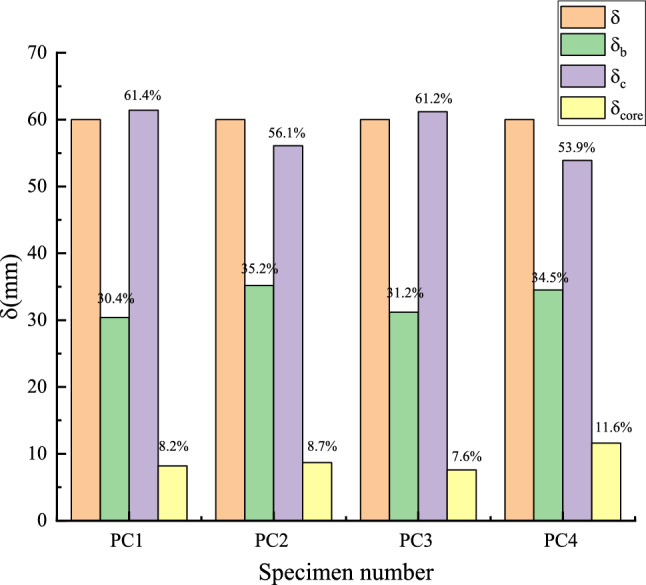
The deformation composition of each node.

Figure 15 shows the deformation ratio of column top caused by various deformations of joint specimens. It can be seen that under the same loading displacement, the deformation values of the joint beams with the same prestress values are basically the same, and the column deformation values of the joints PC1 and PC3 with smaller precast beam deformation are larger. At the same time, it can also be found that the deformation of the joint area with high prestress value and L-shaped steel is also small, indicating that the setting of L-shaped steel can also effectively reduce the deformation of the core area of the protection joint.

## Establishment of the restoring force model

### Establishment of trilinear restoring force model

**Figure 16:**
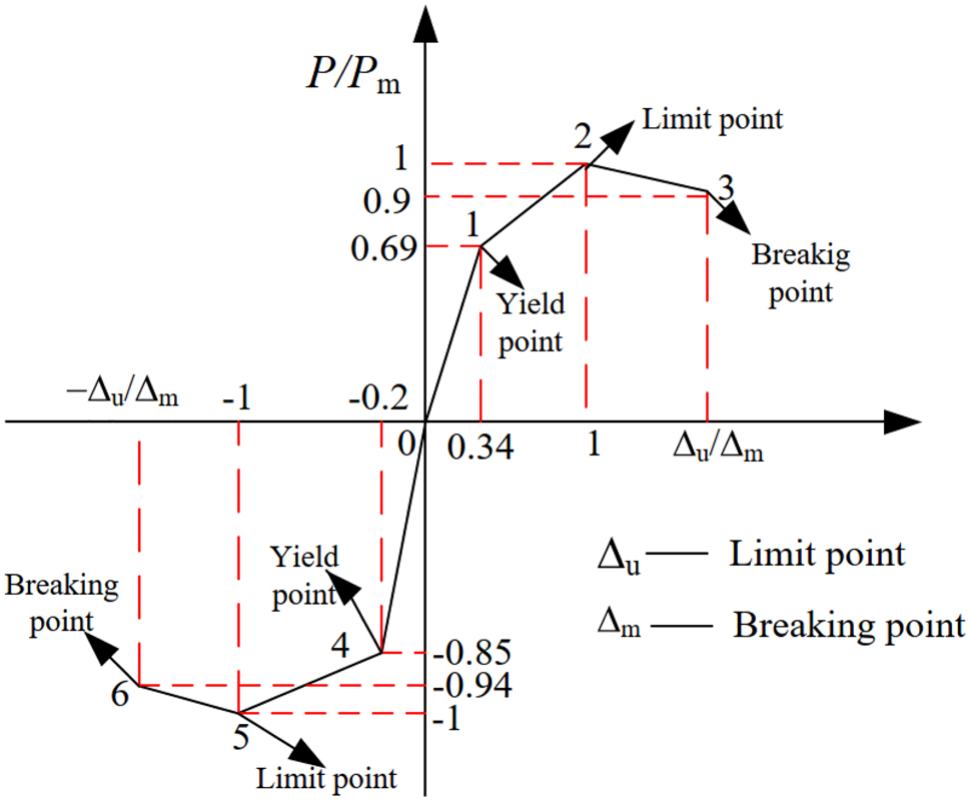
Trilinear skeleton curve determined by experimental regression.

**Figure 17:**
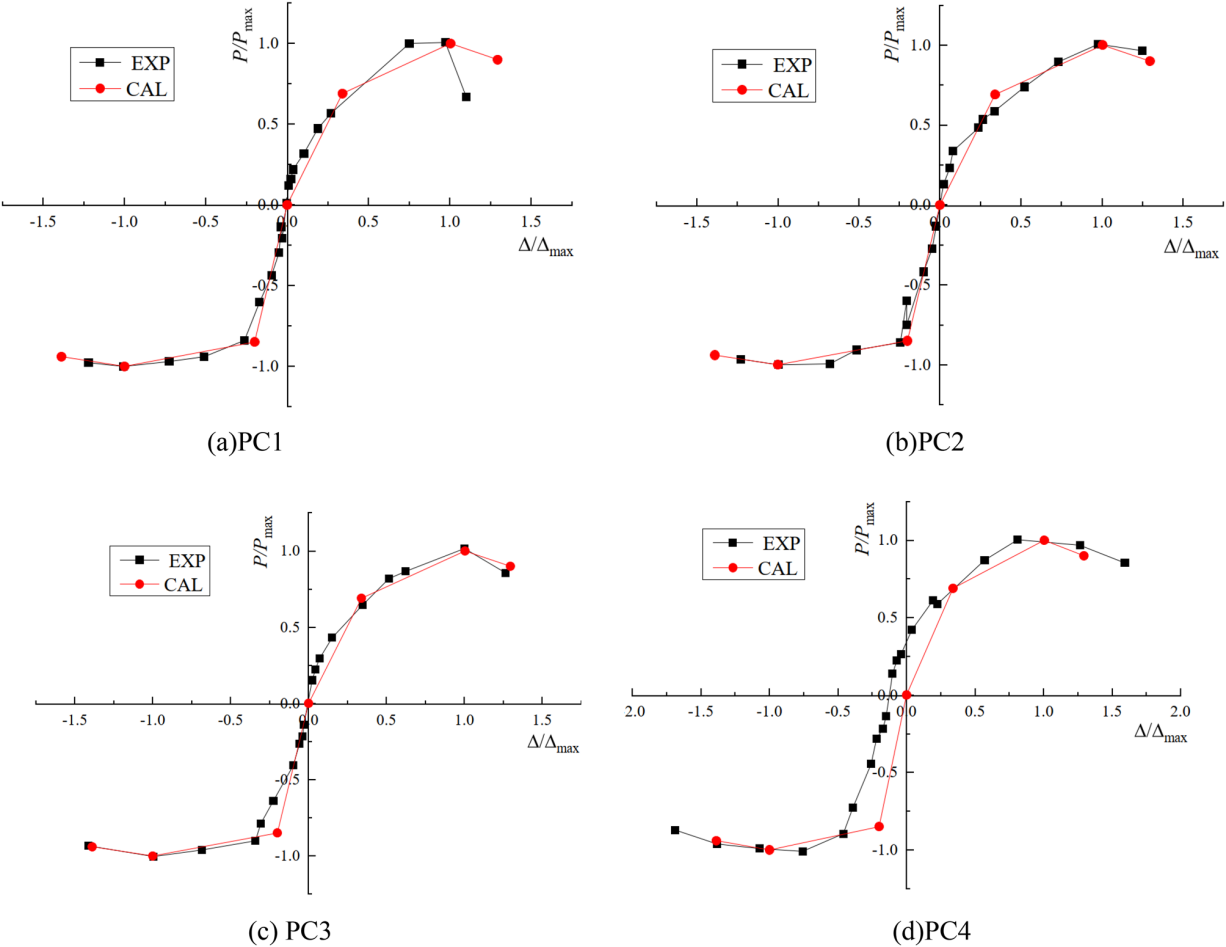
Comparison between theoretical value and experimental value.

This study adopts a widely used stiffness-degraded trilinear model corresponding to the polyline model, which is commonly used in practical engineering^25–28^, as shown in Fig. 16. The critical points of the model mainly include the yield point and the peak point, which correspond to the values of yield strength, peak strength, yield deformation, and peak deformation, respectively. In the restoring force model, the representative points of each stage are determined by experimental regression method. The comparison between the trilinear skeleton curve obtained by regression and the skeleton curves obtained by the test is shown in Fig. 17.

Through Fig. 17, it can be found that the three-line restoring force model proposed in this paper can better reflect the various stress stages of the joint, and it is in good agreement with the experimental results.

### Establishment of the finite element model

Beam and column concrete usually adopts solid element. According to the characteristics of concrete specimens, this paper selects linear reduction integral unit C3D8R. The element has the following advantages: (1) The displacement solution is more accurate; (2) the distortion of the grid has little effect on the accuracy of the analysis; (3) Shear self-locking is not easy to occur under bending load^29–31^.

Steel usually uses truss elements. The reason is that the truss is a member connected by hinges at the end of the bar beam, which is generally mainly subjected to axial tension or pressure, while the steel bar in the joint member is subjected to axial force, and the two characteristics are consistent, so the steel bar unit adopts T3D2.

**Figure 18:**
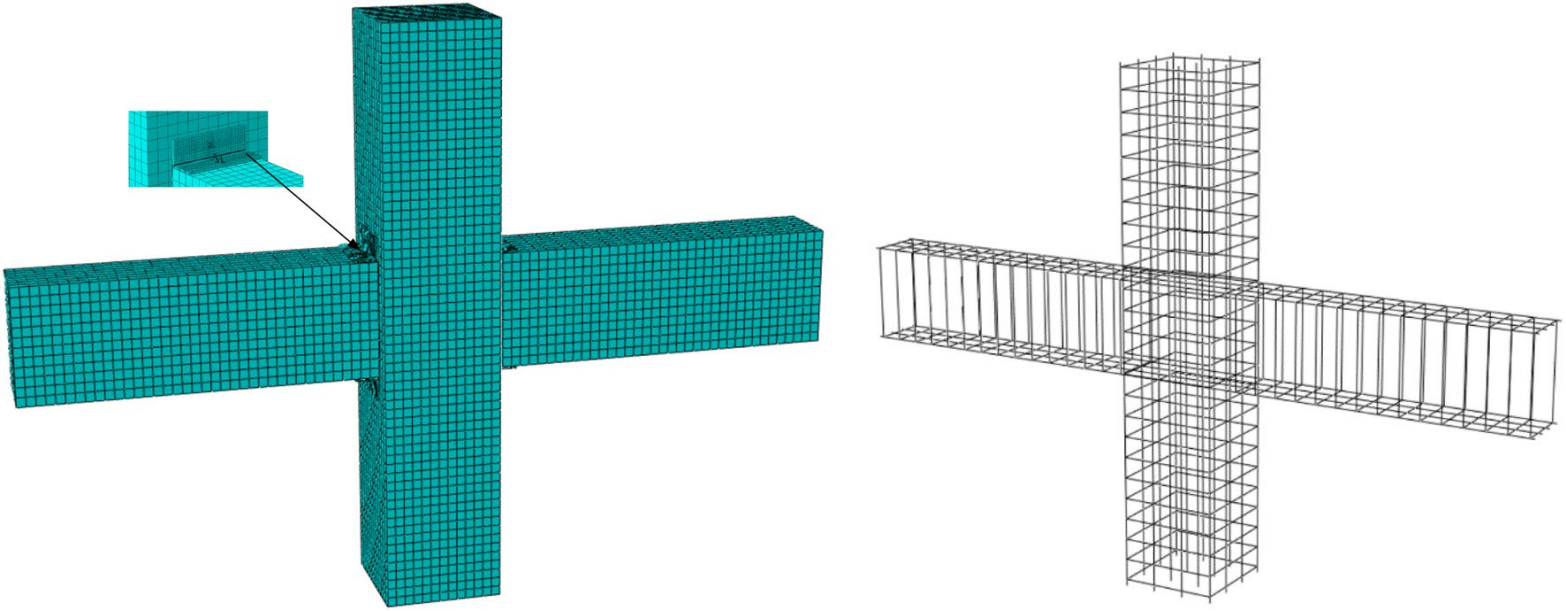
Finite element analysis model.

Through the constitutive relationship of concrete and steel bar, the selection of element type and the setting of boundary conditions, the finite element model of beam-column joints of steel strand anchored assembled concrete frame is established. The node model after meshing is shown in Fig. 18.

### Finite element analysis results

**Figure 19:**
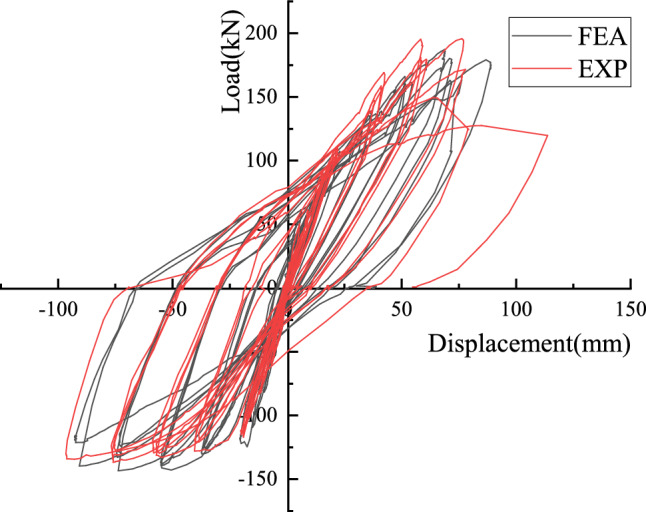
A comparison of finite element calculation and test results.

The finite element model of PC1 was established by the above method, and low cyclic loading was applied to it. The calculation results are shown in Fig. 19.

It can be found from Fig. 19 that the ultimate load of the joints calculated by the finite element method is larger than the test value. The main reason is that the finite element calculation is often too idealistic and does not take into account some gaps in the test process and some defects in the process of component processing. However, the overall calculation results are in good agreement with the experimental results. The overall error is controlled within 10%, which can accurately reflect the mechanical properties of the joints. The finite element method can be used to further analyze the joints.

## Conclusions

This study has improved the connection based on the previous research, the seismic performance of the connections was studied, and the following conclusions were obtained through the research.

1. Using post-tensioned prestressed precast beam-column splicing, its carrying capacity can meet the requirements, and the setting of L-shaped steel can improve the energy consumption of nodes.
2. Setting L-shaped steel at the beam-column joint can effectively improve the ultimate bearing capacity of the joint, and also help to reduce the damage degree of the joint area.
3. By using of post-tensioned prestress to install prefabricated beams and columns can effectively enhance the bearing capacity of the joints and increase the deformation recovery ability of the joints.
4. The finite element analysis method in this paper can accurately predict the mechanical properties of post-tensioned prestressed splice joints, and improve the effective calculation method for further analysis of joints.
5. The size of the prestress value has a significant impact on the bearing capacity and stiffness of the joints, so the size of the prestress value should be controlled when the precast members are assembled.

## Data Availability

Data cannot be shared openly but are available on request from correspondence author.
